# Statistical Learning Model of the Sense of Agency

**DOI:** 10.3389/fpsyg.2020.539957

**Published:** 2020-10-14

**Authors:** Shiro Yano, Yoshikatsu Hayashi, Yuki Murata, Hiroshi Imamizu, Takaki Maeda, Toshiyuki Kondo

**Affiliations:** ^1^Division of Advanced Information Technology & Computer Science, Tokyo University of Agriculture and Technology, Tokyo, Japan; ^2^Biomedical Science and Biomedical Engineering, School of Biological Science, University of Reading, Berkshire, United Kingdom; ^3^Department of Psychology, The University of Tokyo, Tokyo, Japan; ^4^Department of Neuropsychiatry, Keio University School of Medicine, Tokyo, Japan; ^5^Center for Psychiatry and Behavioral Science, Komagino Hospital, Tokyo, Japan

**Keywords:** sense of agency, statistical learning, online learning, Bayes' rule, stochastic gradient descent

## Abstract

A sense of agency (SoA) is the experience of subjective awareness regarding the control of one's actions. Humans have a natural tendency to generate prediction models of the environment and adapt their models according to changes in the environment. The SoA is associated with the degree of the adaptation of the prediction models, e.g., insufficient adaptation causes low predictability and lowers the SoA over the environment. Thus, identifying the mechanisms behind the adaptation process of a prediction model related to the SoA is essential for understanding the generative process of the SoA. In the first half of the current study, we constructed a mathematical model in which the SoA represents a likelihood value for a given observation (sensory feedback) in a prediction model of the environment and in which the prediction model is updated according to the likelihood value. From our mathematical model, we theoretically derived a testable hypothesis that the prediction model is updated according to a Bayesian rule or a stochastic gradient. In the second half of our study, we focused on the experimental examination of this hypothesis. In our experiment, human subjects were repeatedly asked to observe a moving square on a computer screen and press a button after a beep sound. The button press resulted in an abrupt jump of the moving square on the screen. Experiencing the various stochastic time intervals between the action execution (button-press) and the consequent event (square jumping) caused gradual changes in the subjects' degree of their SoA. By comparing the above theoretical hypothesis with the experimental results, we concluded that the update (adaptation) rule of the prediction model based on the SoA is better described by a Bayesian update than by a stochastic gradient descent.

## 1. Introduction

The sense of agency(SoA) is the subjective awareness about “the experience of controlling one's own motor acts and, through them, the course of external events” (Haggard, 2017). The SoA is regarded as a fundamental role in maintaining continuous self-consciousness (Gallagher, 2000). Normally, we are unaware of the existence of the SoA because it exists behind our daily actions. An explicit awareness of the decreased SoA arises as a result of a striking conflict of expectations, i.e., a mismatch between the intended and the actual result of an action (Haggard, 2017).

The SoA has been studied in various disciplines, ranging from psychiatry (Maeda et al., 2012, 2013) to human computer interaction (HCI) domains and brain computer interface (BCI) domains (Coyle et al., 2012; Minohara et al., 2016; Cornelio Martinez et al., 2017). Measuring and understanding the SoA plays an important role in these domains. Disruptions in the SoA in movement disorders have major implications for quality of life (Haggard, 2017). The SoA can be the indicator for identifying the user's experience for an appropriate sense of operation in HCI and BCI domains, as the SoA is associated with the awareness of the response of one's own action.

**Figure 1:**
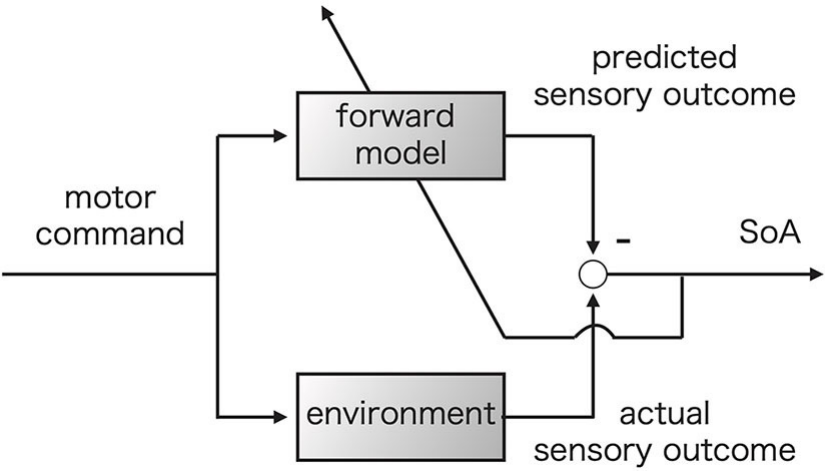
Comparator model (Wolpe and Rowe, 2014). The motor command is fed into the forward model and environment simultaneously. The upper left arrow represents the update of the forward model based on the prediction error to minimize the prediction error (Synofzik et al., 2008).

Previous researchers have assumed that there should exist at least two different generative processes of the SoA: prospective and retrospective processes (Wolpe and Rowe, 2014; Haggard, 2017). The comparator model, which is our focus in this research, corresponds to the former, while the apparent mental causation model corresponds to the latter (Wegner and Wheatley, 1999). Figure 1 shows a schematic picture of the comparator model. The comparator model was originally proposed to describe the process of executing motor controls (Figure 1) (Wolpert et al., 1995; Blakemore et al., 2001). For this process, motor command causes actual bodily dynamics and changes in the external environment. In Figure 1, these changes are represented by the environment block. On the other hand, it is assumed that the motor command also drives the forward model which simulates the dynamics of the body. The actual sensory feedback caused by environmental change and the predicted outcomes are compared following action execution. In the comparator model, the SoA is assumed to be generated only if the sensory feedback and the predicted outcomes match. Any mismatch, i.e., a prediction error, is believed to reduce the SoA (Synofzik et al., 2008; Haggard and Chambon, 2012; Haggard, 2017). Some research assumes that the forward model would be updated to minimize the prediction error (Synofzik et al., 2008; Haggard, 2017). The upward left arrow in Figure 1 represents this update.

Various types of mathematical modeling approaches have been applied in the cognitive science field (Sun, 2008). One of the advantages of the mathematical modeling approach is that it enables the study of implications of the attractive hypotheses with deep insight (McClelland, 2009). While this approach would definitely help the SoA studies, to the best of our knowledge, very few studies have proposed a mathematical formulation of the generative process of the SoA except for the work by Moore and Fletcher (2012) and Legaspi and Toyoizumi (2019). Legaspi and Toyoizumi (2019) proposed a concrete and analyzable formula for the “Bayesian cue integration model” proposed by Moore and Fletcher (2012), which had been described only in the abstract formula. In their model, cue integration corresponds to multi-modal sensory integration, e.g., audiovisual integration. As noted by the authors, their hypothesis is that Bayesian cue integration is the “general principle” behind the SoA generation. One of the important contributions by Legaspi and Toyoizumi (2019) was a proposal of a quantitative definition for the SoA. According to our understanding of their work, at least three important issues exist regarding their model to be discussed.

1. No evidence was shown for their assumption that humans solve the cue integration problem by Bayesian inference. Other well-known solution methods exist for the cue integration problem such as stochastic gradient descent (Morency and Baltrušaitis, 2017).
2. The comparator model does not consider multimodality as the essential assumption. The comparator model tells us that we can sense the SoA even from a single-modal sensory signal alone.
3. They proposed the maximum value of the posterior distribution as a possible candidate for the SoA. This proposal does not fit the comparator model, as the candidate value does not correspond to coincidence between the prediction and the actual outcome.

Many researchers have assumed that Bayesian inference is the fundamental and leading principle algorithm in cognitive brain function (Wolpert and Ghahramani, 2000; Körding, 2008; Sanborn et al., 2010; Pouget et al., 2013; Penny, 2015; Legaspi and Toyoizumi, 2019), known as a “Bayesian coding hypothesis” or a “Bayesian brain hypothesis” (Knill and Pouget, 2004). Gershman (2019) summarizes the hypothesis as follows: (1) the brain is equipped with an internal model, i.e., the likelihood function and prior distribution, and (2) Bayes' conditionalization or its approximations work to update the prior distribution. As we show below, Bayesian inference is only one of many solution methods for some inference problems.

One of the important criticisms for the Bayesian hypothesis in cognitive science is that the researchers blindly accept the hypothesis as the standard principle, i.e., the superiority of the Bayesian inference is rarely verified with respect to other inference rules (Bowers and Davis, 2012; Colombo et al., 2018)Pearl (1988, Chapter 2) (Halpern, 2017, Chapter 3). In particular, there are various proofs that Bayesian inference can be derived from general inference rules, i.e., that Bayesian inference is simply a special case of these rules. While the product rule of the probability is definitely the well-known mathematical foundation of Bayes' theorem (Griffiths et al., 2008), there are at least three theoretical foundations known as Kullback's principle of minimum cross-entropy (MINXENT), i.e., the principle of minimum discrimination information (Shore and Johnson, 1980; Shu-Cherng and Tsao, 2001; Rao, 2011; Halpern, 2017, Chapter 3), the information conservation principle (Zellner, 1988, 2002; Soofi, 2000), and the mirror descent algorithm (Warmuth, 2006; Dai et al., 2016). As shown in Fang et al. (1997), the Bayesian inference can be derived from the MINXENT. The other important inference rules such as the maximum entropy principle (MAXENT) can be derived from the MINXENT (Shu-Cherng and Tsao, 2001). Zellner (2002) introduced a concept of the information processing rule, i.e., the rule that transfers prior information and current information into posterior information. Zellner showed that Bayes' theorem can be viewed as the optimal information processing rule under some specific constraint conditions (the information conservation principle). Despite the fact that it is possible to create numerous mathematical models that can potentially explain certain known phenomena, Bayesian inference was not explored in comparison with other candidates. Thus, in this study, we made a comparison of the Bayesian inference with the stochastic gradient descent.

In the first half of this paper, we describe a systematic derivation for the proposed mathematical model of the SoA on the basis of the comparator model , which is related to the prospective generative process of the SoA. We propose a perspective that the SoA is equal to the likelihood value for a given observation. Then, by focusing on the optimization algorithm to maximize the likelihood function, we introduce a perspective that stochastic gradient descent (SGD) (Bishop, 2006; Morency and Baltrušaitis, 2017, Chapter 5.2.4) is the alternative algorithm to the Bayes rule. This is because both algorithms are equally derived from the mirror descent algorithm (Warmuth, 2006; Dai et al., 2016). The difference between the two algorithms exists only in the constraint to the mirror descent algorithm. Bayes' rule uses the Kullback-Leibler divergence (KL divergence), while the SDG uses the Euclid distance as a constraint to the mirror descent algorithm. The stochastic gradient descent method can also be derived from the mirror descent (Bubeck, 2017, Chapter 6.1). If we adopt the Euclid distance as a constraint to the mirror descent algorithm, it follows the SGD. As both the KL divergence and the Euclid distance are essential components in statistical learning, we propose that it is reasonable to compare the SGD with the Bayes' rule. One of the most important ideas in this paper is that these two algorithms predict different behaviors of learners in an online learning problem setting. By focusing on the difference in the observed behavior, we can confirm which algorithm is more suitable for the explanation of the experimental data. In this paper, we do not provide the details of the mirror descent algorithm but refer to (Bubeck, 2017, Chapter 6.1) and (Warmuth, 2006) for further information on this subject.

In the second half of this paper, we validate whether the learning process of human subjects is based on the Bayes' rule or the stochastic gradient descent by performing participant experiments. The experimental task assigned to the subjects was a sense of agency task (Keio method) in which the subjects were asked to report the perception of the SoA during the repeated sequence of intentional action and the observation of resulting target motion (Maeda et al., 2012, 2013). For each trial in the sense of agency task, the presence or absence of the SoA was reported in response to a question. Our objective in performing the experiments was to measure the degree of the SoA that was gradually changed during the task and to determine whether this was caused by the Bayesian inference or the SGD algorithm. Although it had been reported by previous studies that the SoA adapts over several repeated experiences (Leotti et al., 2015), there are only a few studies that clarify the mechanism behind this adaptation (Legaspi and Toyoizumi, 2019; Di Plinio et al., 2020). We propose an indicator that can be used to test the hypothesis obtained from the proposed mathematical model, and we analyze the gradual changes of the perception of the SoA during the task.

This paper consists of three topics: First, we propose a novel mathematical model for the generative process of the SoA based on the comparator model. Second, we derive a scientifically testable hypothesis from the proposed model. Third, we verify our hypothesis of the generative process of the SoA by participant experiments.

The following is an explanation of the terms used in this paper. The term “statistical learning” is a technical term in cognitive science referring to the ability to extract general rules from a series of observations over time (Santolin and Saffran, 2018), carrying out the implications of some automatic learning processes (Schapiro and Turk-Browne, 2015). The term “forward model” is replaced by the term “predictive distribution” (Wolpert and Ghahramani, 2000) or “statistical model” conditioned by one's own action. As explained in Nguyen-Tuong and Peters (2011, section 2.1.1), the term “forward model” often means a deterministic prediction model. The terms "statistical model" and “predictive distribution” are used to emphasize the aspect of probabilistic models. The differences between these terms are described in the next section.

## 2. Mathematical Models and Hypothesis

### 2.1. The Sense of Agency and the Learning Process

#### 2.1.1. Likelihood Value as the Sense of Agency

In the following section, we explain our perspective regarding the mathematical formulation. We consider the situation where a human subject selects a specific action $a_{k}\in A$ and then observes some realized *i*.*i*.*d*.-values $X_{k}\in X$. The parameter *k* indicates the number of trials (*k* = 1, …, *K*). The value *X*_*k*_ corresponds to the actual sensory outcome at the *k*-th trial. We assume that the human subject possesses the probability density function *p*(*x*|θ, *a*) to predict the sensory outcomes in the future, which is also called the statistical model. The statistical model is parameterized by the parameter θ ∈ ℝ^*n*^.

We focus on two major representations of the parameter: the deterministic manner θ = θ_*k*_ and the probabilistic manner *p*_*k*_(θ). The subject predicts the results of the action using the predictive distribution *p*_*k*_(*x*|*a*). The distribution with deterministic parameter representation is defined as Equation (1):

(1) $$\begin{array}{r} p_{k}\left(x|a\right): =p\left(x|a,\theta_{k}\right), \end{array}$$

and that with probabilistic parameter representation is Equation (2):

(2) $$\begin{array}{r} p_{k}\left(x|a\right): =\int p\left(x|a,\theta \right)p_{k}\left(\theta \right)d\theta . \end{array}$$

The SoA is based on the degree of coincidence between the actual sensory outcome and its prediction. Thus, we propose the perspective of the SoA being the likelihood value for a given observation. The likelihood value at the *k* step is

(3) $$\begin{array}{r} L_{k}: =p_{k}\left(x=X_{k}|a=a_{k}\right). \end{array}$$

Note that *L*_*k*_ is the scalar-valued stochastic variable because of the stochastically generated observations *X*_*k*_. The likelihood value *L*_*k*_ indicates how likely the observed data occur from the viewpoint of the human subject's current predictive distribution for a given action. This means that the likelihood reflects the degree of the discrepancy between the actual observation and the prediction: a higher likelihood represents a lower discrepancy. The comparator model hypothesizes that the SoA is generated based on the discrepancies between the prediction and actual outcomes (Figure 1). Thus, we consider the likelihood value as the SoA.

#### 2.1.2. Online Learning Algorithms: Bayes' Rule and Stochastic Gradient Descent

Data become available in sequential order in real life. Online learning algorithms are the class of algorithms used to update the predictive distribution every time new sensory data are observed (Hazan, 2016). The predictive distribution is updated by the improvement of θ_*k*_ or *p*_*k*_(θ) toward a more precise one in an online manner. As in the standard problem setting, in this study we assume that the distribution is updated to minimize the negative log-likelihood function *L*(θ):

(4) $$\begin{array}{r} L_{k}\left(\theta \right)=-\log p\left(X_{k}|\theta ,a_{k}\right). \end{array}$$

Note that *L*_*k*_ in Equation (3) and *L*_*k*_(θ) in Equation (4) are different objects. *L*_*k*_(θ) is a function of θ.

This section is structured as follows. First, we will introduce a brief review on a derivation of the Bayes' rule (Equations 5–9). Next, we will introduce the stochastic gradient descent algorithm (Equations 10–11).

**Figure 2:**
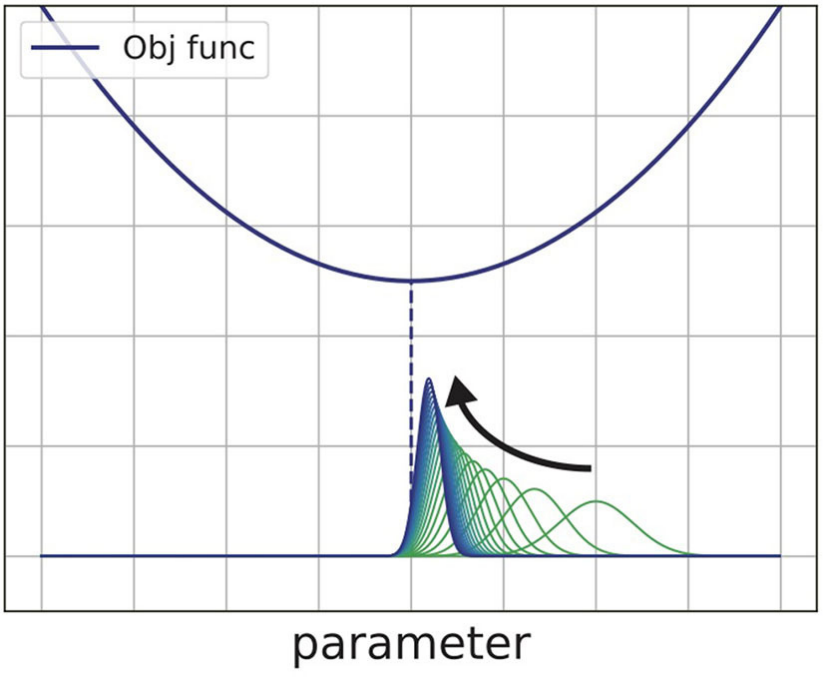
Schematic view of the exponentiated gradient method. This method repeatedly updates the distribution of the parameter to find the minimum value of the objective function.

We will begin by introducing the derivation that states that the Bayes' rule is an algorithm for the minimization of problems. The fact that the Bayes' rule can be derived as a minimization algorithm is useful to understand that this rule is only one of the options for an optimization algorithm. There are two prominent representations of θ that can be used to minimize (Equation 4). One is to search for the optimal θ directly. The other is to search the optimal probability density function *p*(θ) which minimizes the expected loss ∫*p*(θ)*L*_*k*_(θ)*dθ*. The former corresponds to the stochastic gradient descent and the latter corresponds to the Bayes' rule. Equation (5) belongs to the latter, representing an iterative algorithm for the expected loss minimization problem (Beck and Teboulle, 2003):

(5) $$\begin{array}{r} p_{k+1}\left(\theta \right)=\arg\underset{p\in P}{\min}\left\{\int p\left(\theta \right)L_{k}\left(\theta \right)d\theta +\beta_{k}\text{KL}\left[p\left(\theta \right)|p_{k}\left(\theta \right)\right]\right\}, \end{array}$$

where β_*k*_ > 0. P is the set of probability density functions. KL[*p*(θ)|*p*_*k*_(θ)] is called the KL divergence, which is a measure of the similarity of two probability distributions. The posterior distribution *p*_*k*+1_(θ) becomes the balanced solution between the prior *p*_*k*_(θ) and the most optimal solution because the KL divergence works as a penalty function. It is notable that Equation (5) is equivalent to Equation (6), which is also known as the normalized exponentiated gradient algorithm, multiplicative weight algorithm, entropic mirror descent algorithm, and so on (Beck and Teboulle, 2003; Shalev-Shwartz, 2011).

(6) $$\begin{array}{r} p_{k+1}\left(\theta \right)=\frac{1}{Z}\exp\left(-\beta_{k}^{-1}L_{k}\left(\theta \right)\right)p_{k}\left(\theta \right), \end{array}$$

where *Z* is the normalization factor $Z=\int \exp\left(-\beta_{k}^{-1}L_{k}\left(\theta \right)\right)p_{k}\left(\theta \right)d\theta$. The equivalence is shown by solving the right-hand side of Equation (5) (Beck and Teboulle, 2003). In Equation (6), prior distribution *p*_*k*_(θ) is weighted in responding to the value of objective function *L*_*k*_(θ). Figure 2 shows the process of minimizing some quadratic objective function with Equation (5). As that equation shows, the algorithm finds the posterior distribution near the prior distribution at each step. The probability distribution *p*_*k*_(θ) finally converges to the Dirac's delta function which surrounds the smallest value of the objective function.

Bayes' rule can be obtained by substituting the negative log-likelihood function into Equation (6) (Warmuth, 2006).

(7) $$\begin{array}{r} p_{k+1}\left(\theta |X\right)\propto p{\left(X_{k}|\theta \right)}^{\frac{1}{\beta_{k}}}p_{k}\left(\theta \right). \end{array}$$

It is important to note that a similar equation is obtained in an iterative manner:

(8) $$\begin{array}{r} p_{k+1}\left(\theta |X_{k}\right)\propto p{\left(X_{k}|\theta \right)}^{\frac{1}{\beta_{k}}}p_{k}\left(\theta |X_{k-1}\right)\propto \cdots \propto \overset{k}{\underset{l=1}{\prod }}p{\left(X_{l}|\theta \right)}^{\frac{1}{\beta_{l}}}p\left(\theta \right) \end{array}$$

where $X_{k}=\left\{X_{1},...,X_{k}\right\}$. In this procedure, the prior distribution is updated with each observation *X*_*k*_. This is the online learning algorithm (Shalev-Shwartz, 2011). In the Bayesian framework, the predictive distribution at each step is defined as

(9) $$\begin{array}{r} p_{k}\left(x|X_{k-1}\right)=\int p\left(x|\theta \right)p_{k}\left(\theta |X_{k-1}\right)d\theta , \end{array}$$

so the likelihood of the observation *X*_*k*_ is $p_{k}\left(X_{k}|X_{k-1}\right)$.

Next, we introduce the stochastic gradient descent (SGD). SGD is also known as an online algorithm that directly updates θ ∈ ℝ^*n*^ such that

(10) $$\begin{array}{rcl} \theta_{k+1} & = & \theta_{k}-{\eta_{k}}^{-1}g\left(\theta_{k}\right), \end{array}$$

(11) $$\begin{array}{rcl} g\left(\theta \right) & = & -\nabla_{\theta }\log p\left(X_{k}|\theta \right), \end{array}$$

where $\eta_{k}>0,\overset{\infty }{\underset{k=1}{\sum }}\eta_{k}^{-1}=\infty$, and $\overset{\infty }{\underset{k=1}{\sum }}\eta_{k}^{-2}<\infty$ (Bishop, 2006, Chapter 5.2.4). The parameter is updated by each additional observation. In this model, the parameter θ_*k*_ holds the prior knowledge. Since the predictive distribution is *p*(*x*|θ_*k*_), the likelihood is represented as *p*(*X*_*k*_|θ_*k*_). Both the entropic mirror descent Equation (5) and the stochastic gradient descent Equation (10) are equally derived from the mirror descent algorithm (Beck and Teboulle, 2003), and the Bayes' rule can be derived from the entropic mirror descent as described by Equation (7). This indicates that it is natural to compare the Bayes' rule Equation (7) and stochastic gradient descent as expressed in Equations (10) and (11).

Our major argument in this section is that we should test the Bayesian hypothesis against another method such as the stochastic gradient descent. In our view, Bayes' rule is not an oracular theorem but just one of the optimization algorithms for the likelihood maximization problem. We tested the Bayesian hypothesis against the stochastic gradient descent, which is reported below.

### 2.2. Scientifically Testable Hypothesis for the SoA Attribution Task

The type of learning algorithm, for instance Bayes' rule or stochastic gradient descent, has strong effects on the learning curve. As we will see in this section, these methods show the different learning curves of each. Thus, the analysis of the learning curve enables us to test whether the actual learning process is based on the Bayes' rule or the stochastic gradient descent.

We consider the situation in which humans are exposed to an environment in which the consequences of their actual actions are not free from a probabilistic temporal bias. The following description assumes the experimental protocol described in the experimental setup section in the second half of this paper (cf. section 2.3). There exists a temporal bias *x* ∈ ℝ [ms] between the timing of “press button (action)” and “the occurrence of the event (the actual outcome of an action).” We used a one-dimensional Gaussian distribution as a model of human subjects' internal representation regarding the distribution of the temporal bias *x* for the mathematical simplicity of the data analysis. Specifically, the subject infers the mean value of the Gaussian distribution and does not infer its standard deviation. Note that this simple assumption requires future validation.

Each subject possesses the statistical model *p*(*x*|μ), which is a one-dimensional Gaussian as expressed in Equations (12) and (13).

(12) $$\begin{array}{rcl} p\left(x|\mu \right) & \propto & \exp\left(L\left(x,\mu \right)\right) \end{array}$$

(13) $$\begin{array}{rcl} L\left(x,\mu \right) & = & -\frac{{\left(x-\mu \right)}^{2}}{2\sigma^{2}}, \end{array}$$

where μ ∈ ℝ and σ ∈ ℝ^+^. The symbol θ used in the previous section corresponds to the symbol μ in this section.

The statistical model is used for predicting the temporal bias *x* between the button press and actual outcome. There are various types of human subjects, some trying to make exact predictions and some trying to make approximate predictions. The individual difference is reflected by the parameter σ.

The statistical model *p*(*x*|μ) is updated by the improvement of μ or *p*(μ) toward a more precise one. By applying Equations (10) and (11), we derive the learning dynamics of the SGD-type subject in section 2.2.1. By applying Equation (7), we derive the learning dynamics of the Bayesian-type subject in section 2.2.2. Finally, we compare these dynamics and provide the scientifically testable hypothesis in section 2.2.3.

#### 2.2.1. Learning Dynamics of the SGD-type Subject

Assume that the human subject observes the i.i.d. temporal biases *X*_*k*_ [ms] at each trial *k*.

By applying the SGD algorithm (Equations 10, 11) to the statistical model in Equations (12) and (13), the learning dynamics of the SGD-type subject are derived as Equation (14).

(14) $$\begin{array}{r} \mu_{k+1}=\mu_{k}+\frac{\eta_{k}^{-1}\left(X_{k}-\mu_{k}\right)}{\sigma^{2}}. \end{array}$$

**Figure 3:**
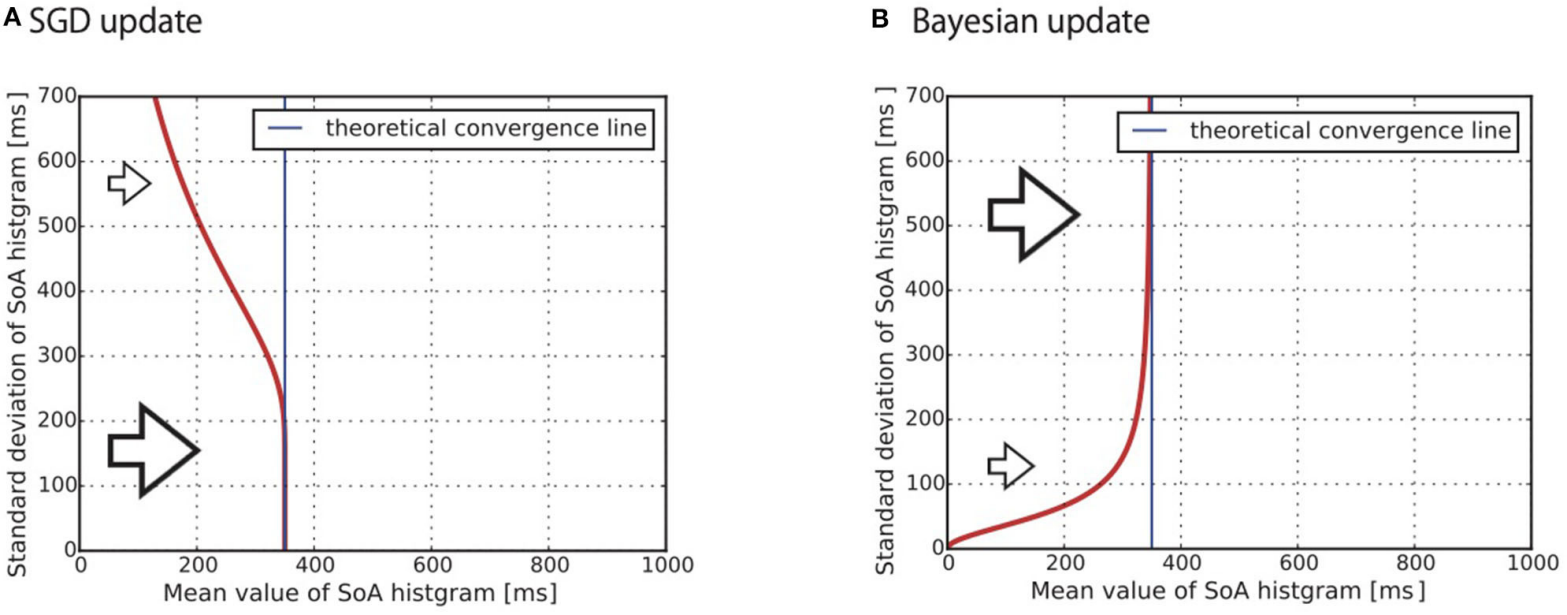
Theoretical prediction for convergence to the mean value of the SoA from **(A)** Equation (10) and **(B)** Equation (17). The standard deviation (SD) of the predictive distribution causes a difference in the learning speed. **(A)** Stochastic gradient descent: the smaller SD subject shows faster convergence **(B)** Bayesian update: the larger SD subject shows faster convergence.

It is important to note that the parameter σ determines the learning speed in Equation (14). More precisely, it predicts that a subject with a larger σ shows a slower learning speed. Figure 3A shows a snapshot of *k*-step of the numerical simulation of Equation (14). The vertical axis shows the σ and the horizontal axis shows the μ. As we previously mentioned, each subject is represented by a different parameter σ. The red line indicates that a subject with a larger σ shows the slower convergence. The blue line is the $\mu =\overset{m}{\underset{i=1}{\sum }}X_{i}$ where Equation (14) converges regardless of the parameter σ. Since the true distribution that generates *X*_*i*_ was assumed to be uniform between –300 and 1,000 ms in this simulation, the equation converges to a mean value 350 ms.

As discussed in section 2.1.1, the predictive distribution for the SGD-type subject is modeled as *p*(*x*|*a*, μ_*k*_) (see Equation 1). If the SGD hypothesis is correct, the predictive distribution *p*(*x*|*a*, μ_*k*_) behaves slower when its standard deviation is larger. This means that the distributions of the likelihood values, i.e., the histogram of the SoA, also behaves in the same way.

#### 2.2.2. Learning Dynamics of the Bayesian-Type Subject

Next, we introduce the learning dynamics of the Bayesian-type subject. We assume that this subject holds the prior distribution described by Equations (15) and (16).

(15) $$\begin{array}{rcl} p_{0}\left(\mu \right) & \propto & \exp\left(\frac{1}{2\sigma_{0}^{2}}G_{0}\left(\mu \right)\right) \end{array}$$

(16) $$\begin{array}{rcl} G_{0}\left(\mu \right) & = & -\mu^{2} \end{array}$$

In this model, the individual difference is reflected by both σ and σ_0_.

By applying Equation (7) to the model expressed in Equations (12), (13), (15), and (16), the learning dynamics of the Bayesian-type subject are derived as Equation (17).

(17) $$\begin{array}{r} p_{k+1}\left(\mu \right): =p\left(\mu |X_{k+1}\right)\propto \exp\left(\overset{k}{\underset{i=1}{\sum }}\frac{1}{\beta_{i}}L\left(X_{i},\mu \right)+\frac{1}{2\sigma_{0}^{2}}G\left(\mu \right)\right) \end{array}$$

The posterior distribution gradually converges to $\underset{t\to \infty }{\lim}p\left(\mu |X_{t+1}\right)\propto \exp\left(\underset{t\to \infty }{\lim}\overset{t}{\underset{i=1}{\sum }}\frac{1}{\beta_{i}}L\left(X_{i},\mu \right)\right)$ as the subject obtains observations. The parameters σ and σ_0_ determine the learning speed in Equation (17). The way σ works is the same as in the SGD case: a larger σ results in a slower learning speed. On the other hand, a larger σ_0_ results in a faster learning speed. The smaller σ_0_ becomes, the greater the importance of *G*(μ); this makes the posterior distribution difficult to shift from the prior distribution. This is a major property of Bayesian inference that we would like to highlight in this study. Due to this property, the learning dynamics of the Bayesian-type subject can behave as shown in Figure 3B. A subject who has a larger standard deviation of the predictive distribution shows faster convergence to the convergence line. Specifically, the larger standard deviation of the prior distribution σ_0_ is the key factor causing this behavior. Figure 3B shows a snapshot of the numerical simulation of Equation (17). The vertical axis shows the standard deviation of the predictive distribution. It is important to note that the SGD hypothesis cannot explain the phenomenon that the curve is rising to the right shoulder as shown in Figure 3B.

As discussed in section 2.1.1, the predictive distribution for the Bayesian-type subject is modeled as ∫*p*(*x*|*a*, μ)*p*_*k*_(μ)*dμ* (see Equation 2). The discussion in this section indicates that the distribution of the likelihood values, i.e., the histogram of the SoA, responds faster when its standard deviation is large.

#### 2.2.3. Proposed Hypothesis for the SoA Attribution Task

To summarize sections 2.2.1 and 2.2.2, our theory suggests the following:

1. An experimentally measured histogram of the SoA would show the learning behavior in response to the time interval between motor execution and the actual sensory outcome.
2. If the experimentally measured histogram of the SoA with a larger standard deviation were to show faster convergence in the learning processes, it would be supportive evidence for Bayesian learning.

In the second half of this paper, we demonstrate two points through experimentation: (1) that the histogram of the SoA shows the learning behavior, and (2) that the learning behavior is due to Bayesian inference.

### 2.3. Experimental Protocol to Quantify the Sense of Agency

In previous SoA research, there are two types of tasks for measuring the SoA. One is an explicit measure of the SoA in which subjects verbally report how much they feel a sense of control over external events (an agency attribution task). The other is an implicit measure of the SoA, in which the SoA is evaluated by the “intentional binding effect” (Haggard, 2017), which refers to the subjective binding in time of voluntary actions to their sensory consequences.

In this study, to investigate the adaptation process of a prediction model on the SoA, we used our original agency attribution task (Keio method). A task was established in which human subjects were asked to report their SoA based on their perception of the causal relationship between an intentional action and the visual outcome (Maeda et al., 2012, 2013). The experimental protocol in this study is based exactly on the protocol described in the studies by Maeda et al. (2012, 2013).

**Figure 4:**
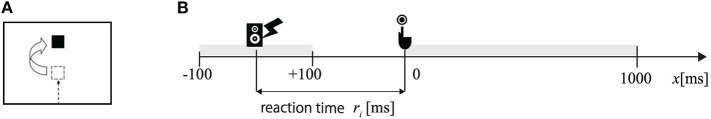
**(A)** Each trial started with a dark computer screen. A square shape appeared at the bottom of the screen and moved straight upwards at a uniform speed (22 [mm/s]). **(B)** The human subjects were instructed to press a button when they heard a beep. When the button was pressed, the square jumped 35 [mm] upward, with various temporal biases. The jump of the square had action-linked conditions and event-prior-to-action (EPA) conditions. In the action-linked conditions, temporal biases were introduced from 0 to 1,000 [ms] in 100-ms increments. In the EPA conditions, the movement of the square was based on the beep and not on the button press, where the movement of the square was programmed to precede the subjects' intentional actions. There were three EPA conditions in which the square jumped 100 [ms] before the beep, at the time of the beep, or at 100 [ms] after the beep. The subjects answered “Yes” or “No” about whether they felt that the square jumped as they intended.

**Figure 5:**
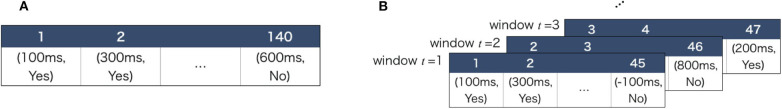
**(A)** The table shows the example result of the Yes/No questions per subject. **(B)** We segmented the table into the window *t* = 1, 2, ...95. Then we calculated the mean value for each histogram.

The experimental stimuli were presented on a 14-inch computer monitor. A 5-mm square shape appeared from the bottom of the screen and moved straight upwards at a uniform speed (22 [mm/s]). The human subjects were instructed to push a button as quickly as possible when they heard a beep. After they pushed the button, the square jumped 35 mm upwards after a random temporal bias, i.e., at [0, 100, 200, 300, 400, 500, 600, 700, 800, 900, 1,000] [ms]. (Figure 4A) Then, they were instructed to respond orally whether they felt that they had caused the square to jump upward as intended by giving a “Yes” or “No” response. A “Yes” response meant that they attributed the jump of the square to their button press, i.e., they felt an SoA during the action. Each condition was conducted 10 times (i.e., 11 conditions × 10 times = 110 trials). In addition to these trials, “event prior to action” (EPA) trials were included in which the square jumped when the beep occurred instead of when the button was pressed. The three EPA conditions were as follows: the square jumped at 100 ms before the beep, at the time of the beep, or at 100 ms after the beep. They were also instructed to respond whether they felt an SoA during these trials. Each EPA condition was also conducted 10 times (i.e., 3 conditions × 10 times = 30 trials). Figure 4B shows the timing at which the temporally biased event occurred. Therefore, we obtained 140 yes/no responses per subject, as represented in Figure 5A.

### 2.4. Participants

Twenty-one healthy volunteers were enrolled in this study (8 males and 13 females). Their mean age was 22.0 ± 1.4SD. years old. They were confirmed to have no psychiatric or neurological disorders. This study was approved by the Ethics Committee at Komagino Hospital and the Tokyo University of Agriculture and Technology. All subjects gave written informed consent prior to participation.

### 2.5. Statistical Analysis

As shown in Figure 5A, we obtained 140 responses per subject. Our purpose of the analysis was to verify the two hypotheses shown in section 2.2.3:

1. The experimentally measured histogram of the SoA would show the learning behavior in response to the temporal bias.
2. If the experimentally measured histogram of the SoA with a larger standard deviation were to show faster convergence in the learning processes, it would be supportive evidence for the Bayesian learning.

#### 2.5.1. Dividing Data by Time-Window

We focused on analyzing the histogram consisting of Yes answers. It is reasonable to assume that the histogram consisting of Yes answers reflects the histogram of likelihood, i.e., the histogram of the SoA. In the following, the term “histogram” refers to the histogram consisting of Yes answers.

To verify these two hypotheses, we first divided the sequence of YES/NO answers using the moving window which consisted of 45 trials (Figure 5B). By dividing the data in this way, it was possible to analyze how the histogram changes over time. We decided on a value of 45 as a criterion to obtain the shape of each of the histograms because a majority of the responses with No would not allow us to analyze the histogram.

#### 2.5.2. Gaussian Curve Fit

By dividing the data into the windows, we obtained 95 histograms per subject (Figure 5B). As noted in section 2.2, we assumed that the subjects employ a one-dimensional Gaussian distribution as a statistical model. This means that the histograms were also assumed to have the shape of a one-dimensional Gaussian distribution, corresponding to the histogram of the likelihood.

We performed the following procedure to estimate the mean values and standard deviations of the histograms:

First, we quantitatively defined the center of each of the bins of the histogram. Regarding the timing of visual stimuli presentation, while no individual differences occurred for the positive temporal biases (0 to 1,000 [ms]), we needed to consider the individual differences due to the reaction time for the negative temporal biases (cf. Figure 1). We calculated the averaged reaction time during the 140 trials for each subject. Then we defined the center of each bin as

(18) $$\begin{array}{r} B_{i}: =\left(-r_{i}-100,-r_{i},-r_{i}+100,0,100,\ldots ,1000\right) \end{array}$$

per subject. Here, *r*_*i*_ > 0 represents the *i*-th subject's averaged reaction time during the 140 trials.

Second, we performed the Gaussian curve fitting to the histograms according to the following procedure: We introduce the notation $\left(B_{i},H_{i}^{t}\right)$ to represent the *i*-th subject's *t*-th window histogram. $B_{i}$ is the 14-dimensional vector that we defined in Equation (18). $H_{i}^{t}\in {\mathbb{N}}^{14}$ is another 14-dimensional vector that consists of the number of the YES answers corresponding to the temporal biases $B_{i}$. We introduce *b*_*i*_(*j*) to represent the *j*-th element of the vector $B_{i}$ (*j* = 1, …, 14). We also introduce $h_{i}^{t}\left(j\right)$ to represent the *j*-th element of the vector $H_{i}^{t}$. Although the standard procedure to estimate the Gaussian distribution is to solve the maximum likelihood problem, we cannot apply this approach in this case. This is because the histogram $\left(B_{i},H_{i}^{t}\right)$ lacks the data corresponding to the three temporal bias domains (−∞, −*r*_*i*_−100], [−*r*_*i*_+100, 0], and [1000, +∞). Instead, we solved a non-linear least square problem such that

(19) $$\begin{array}{r} Z_{i}\left(t\right),\mu_{i}\left(t\right),\sigma_{i}\left(t\right)=\arg\underset{Z,\mu ,\sigma }{\min}\overset{14}{\underset{j=1}{\sum }}{\left(f\left(b_{j};Z,\mu ,\sigma \right)-h_{i}^{t}\left(j\right)\right)}^{2}, \end{array}$$

where *f*(·;*Z*, μ, σ) is the Gaussian function:

(20) $$\begin{array}{r} f\left(x;Z,\mu ,\sigma \right)=Z\exp\left(-\frac{{\left(x-\mu \right)}^{2}}{2\sigma^{2}}\right). \end{array}$$

We applied the Levenberg-Marquardt algorithm^1^ to problem (19) and obtained (μ(*t*), σ(*t*), *Z*(*t*)) which are the estimations of the mean value, the standard deviation, and the scaling factor, respectively.

#### 2.5.3. Learning Curve Analysis

By analyzing (_μ_*i*_(*t*), σ_*i*_(*t*))*t* = 1, …, 95_ obtained from the Gaussian curve fitting, we can clarify how the predictive distribution behaved. The aim of this analysis was to determine whether the learning behavior is occurring for the temporal bias and whether the learning algorithm is the Bayesian or the SGD algorithm. The main idea was to confirm whether the indicators (_μ_*i*_(*t*), σ_*i*_(*t*))*t* = 1, …, 95_ show a similar movement to either Figure 3A or Figure 3B.

We focused on two features of Figure 3. The first feature is convergence toward the “theoretical convergence line.” The red lines gradually converge to the “theoretical convergence line” which is also shown in the figure. The theoretical convergence line is settled as the solution to the KL divergence minimization problem of the true distribution and the statistical model. As we assumed that the statistical model is the Gaussian distribution described by Equations (12) and (13), the line becomes the expectation of the observed values ${\text{E}}_{q\left(x\right)}\left[x\right]≃\frac{1}{K}\overset{K}{\underset{k=1}{\sum }}X_{k}$ where *q*(*x*) is the true distribution of the observed values *X*_*k*_~*q*(*x*) (Wolpert et al., 2013, section 5.1.1). As noted in the previous section 2.5.2, the set of the temporal biases $B_{i}$ is affected by the individually different reaction times *r*_*i*_(*i* = 1, ..., 21). This individual difference makes it difficult to calculate the convergence line $\frac{1}{K}\overset{K}{\underset{k=1}{\sum }}X_{k}$. To approximately estimate the line, we assumed the special case that all subjects equally showed the same reaction time *r*_*i*_ = 200 [ms]. Under this assumption, we calculated $\frac{1}{K}\overset{K}{\underset{k}{\sum }}X_{k}≃350$ [ms]. Note that we adopted the assumption only for the approximate estimation of the line. We did not adopt the assumption for the other types of statistical analysis in this paper. All of the other statistical analyses were achieved using the estimation of the individually different reaction times (cf. section 2.5.2). The approximate estimated line was used only as a guide to confirm whether the data converged to the line. The convergence indicates that the subjects learned the temporal bias.

The second significant difference between the two subfigures Figures 3A,B appears in the slope of the red line. The red line shows a negative slope in Figure 3A. This reflects the implication of Equation (14) that, under the SGD hypothesis, a subject who has a larger standard deviation of the predictive distribution shows the slower convergence to the convergence line (cf. section 2.2.1). In contrast, the red line shows a positive slope in Figure 3B. This reflects the implication of Equation (17) that, under the Bayesian hypothesis, a subject who has a larger standard deviation of the predictive distribution shows faster convergence (cf. section 2.2.2).

We addressed two questions: (1) whether the learning of the temporal bias occurred and (2) whether the learning algorithm is the Bayesian or the SGD algorithm. To answer the first question, we analyzed how the center of the scatter plot (μ_*i*_(*t*), σ_*i*_(*t*)) changed as the number of trials increased. To answer the second question, we investigated whether the slope of the scatter plot was negative or positive, and how the slope changed as the number of trials increased. These analyses were applied to the scatter plots of (μ_*i*_(*t*), σ_*i*_(*t*)) at the group level. To perform a quantitative analysis, we applied a linear regression on each scatter plot using Equation (21).

(21) $$\begin{array}{r} \sigma =b_{t}\left(\mu -a_{t}\right)+c_{t}. \end{array}$$

For the linear regression, we applied the Levenberg-Marquardt algorithm to the least square problem. We obtained a time sequence of the estimated parameters (_*a*_*t*_, *b*_*t*_, *c*_*t*_)*t* = 1, …, 95_, as we have the time sequence of the scatter plots. If the time evolution of the estimated parameter *a*_*t*_ shows convergence to the neighbor of 350 [ms], which is the approximate estimation of the convergence line (see section 2.5.2), it is supportive evidence that the subjects showed learning behavior to the temporal bias. If the estimated slopes *b*_*t*_ are positive at almost all the time steps, it implies that the standard deviation increases as the mean value increases (Figure 3B: Bayes' rule). Then, we rejected the SGD algorithm hypothesis (Figure 3A). We applied the binomial test for the set of the estimated slopes: the null hypothesis was that the observed proportion of the positive slopes *b*_*t*_ > 0 vs the negative slopes *b*_*t*_ < 0 is not different from 0.5. If the hypothesis is rejected, it indicates that positivity or negativity of the slope exists. We set the significance level as α = 0.05.

## 3. Results

**Figure 6:**
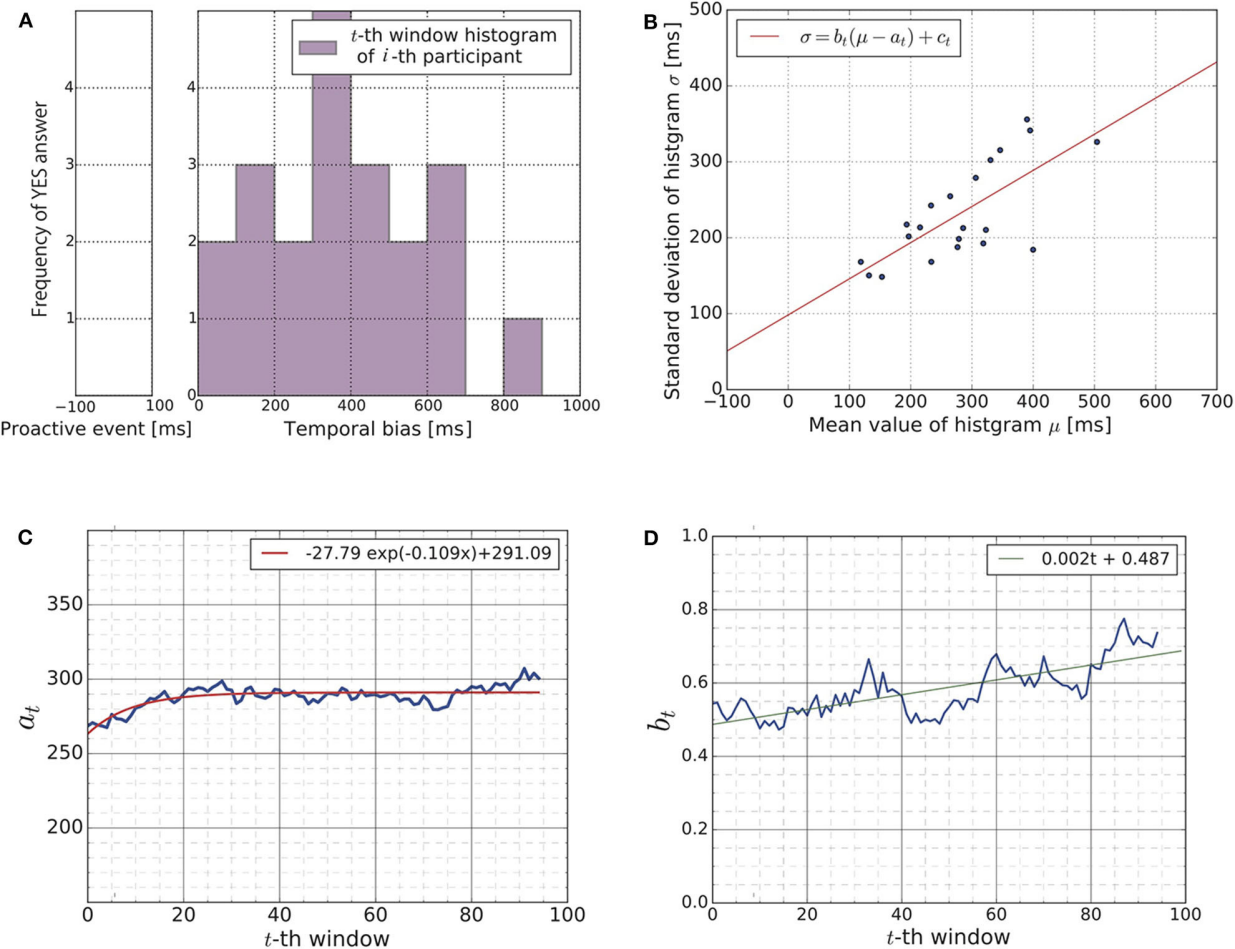
**(A)** The histogram visualizes the distribution of YES answers obtained from a human subject (the *i*-th subject) during a time-window (the *t*-th time-window). The figure shows the result of *i* = 4 and *t* = 1 as an example. We have a total of 95 × 21 histograms as there are 95 time-windows for each subject. To analyze these histograms, we summarized each histogram through the mean value μ_*i*_(*t*) and standard deviation σ_*i*_(*t*) using Gaussian curve fitting. Note that the figure shown here is just one example of these histograms, which indicates that the subject feels most confident around approximately 350 [ms] of temporal bias. **(B)** We summarized the 95 × 21 histograms to the time sequence of the scatter plots. Each scatter plot contains 21 points (_μ_*i*_(*t*), σ_*i*_(*t*))*i* = 1, ..., 21_, as the figure shows. The figure shows a snapshot of the time sequence as an example (*t* = 50). Each blue point represents the mean and the standard deviation of a subject's histogram. The red line shows the result of the linear regression (Equation 21). We focused on the time evolution of the parameters *a*_*t*_ and *b*_*t*_ for our further analysis. **(C)** Time evolution of the parameter *a*_*t*_ showing the gradual convergence of the parameter *a*_*t*_, which indicates that learning occurred during the experiment. **(D)** Time evolution of the parameter *b*_*t*_. This parameter was positive at any given point in time and gradually increased over time.

Figure 6A shows a distribution of YES answers obtained from a subject (the *i*-th subject) during the *t*-th time-window (cf. Figure 5B). We have a total of 95 × 21 histograms, as there are 95 time-windows and 21 subjects. Figure 6A is simply an example of these histograms. Using a Gaussian curve fit (cf. section 2.5.3), we obtained the mean value and the standard deviation (μ_*i*_(*t*), σ_*i*_(*t*)) of each histogram.

Figure 6B shows the scatter plot consisting of 21 points (_μ_*i*_(*t*), σ_*i*_(*t*))*i* = 1, …, 21_ for one of the 95 time-windows. Each point corresponds to subject. The horizontal axis represents the mean value, and the vertical axis represents the standard deviation. Since we have 95 time-windows, we obtained a time sequence of 95 scatter plots. Figure 6B is simply a snapshot of the time sequence. The red line in Figure 6B shows the result of the linear regression Equation (21). We obtained a time sequence of the estimated parameters (_*a*_*t*_, *b*_*t*_, *c*_*t*_)*t* = 1, …, 95_ by applying the linear regression to each of the 95 scatter-plots of (μ_*i*_(*t*), σ_*i*_(*t*)).

Figure 6C shows the time evolution of the parameter *a*_*t*_, i.e., (*a*_1_, …, *a*_95_) (blue line). The red curve in the figure is the exponential regression curve to the time evolution. It mostly converges to approximately 291 [ms] which is close to the average value of the uniform distribution of the temporal bias, which is approximately 350 [ms]. This result is supportive evidence for the existence of the learning process of subjects during the experiment. For the exponential curve regression, the Levenberg-Marquardt algorithm was applied to the least square problem.

Figure 6D shows the time evolution of the parameter *b*_*t*_, i.e., (*b*_1_, …, *b*_95_). As it shows, the parameter *b*_*t*_ was positive and gradually increased across the 95 time-windows. As we explained in section 2.2.3, the positivity of the parameter *b*_*t*_ supports the hypothesis that the updating rule of the subjects is the Bayesian update. As all 95 parameters (*b*_1_, …, *b*_95_) were positive, we applied a binomial test to confirm the statistical significance of the positivity of *b*_*t*_ as explained in section 2.5.3. The null hypothesis was that the observed proportion of the positive slopes *b*_*t*_ > 0 vs. the negative slopes *b*_*t*_ < 0 was not different from 0.5. The null hypothesis was rejected (*p*-value < 2.2*e*^−16^ < 0.05). The 95 percent confidence interval of the ratio regarding the number of negative slopes and positive slopes was (0.00, 0.04). These results indicate that the updating rule of the subjects should be the Bayesian update. For the linear regression shown in Figure 6B, the Leveberg-Marquardt algorithm was applied to the least square problem.

To conclude, our results in Figure 6 suggest that the learning process of the SoA for temporal bias should be described by the update of the predictive distribution in Bayes' rule rather than the SGD algorithm.

## 4. Discussion

The sense of agency is the experience of subjective awareness regarding the initiation and control of one's own actions. The comparator model indicates the process required to generate the SoA, i.e., a mismatch between the prediction and the consequent event simply causes the loss of the SoA. Therefore, discrepancies in timing between the prediction and event hinder the flow of information integration and thus the induction of the SoA (Wegner and Sparrow, 2004; Wegner et al., 2004). This means that the individual lack ownership of the motor actions if the visual stimuli occur after a short time delay (Saposnik et al., 2010). However, through interactions with the environment, humans have a natural tendency to adapt our control ability and to adapt our internal model to predict the environment. Along this line, the adaptive aspects of the SoA toward changes in the surrounding environment have been debated (Leotti et al., 2015; Haggard, 2017; Legaspi and Toyoizumi, 2019; Di Plinio et al., 2020). As pointed out by Di Plinio et al. (2020), understanding the mechanisms behind the generation and adaptation of SoA is an important emerging topic in this field. While Legaspi and Toyoizumi (2019) proposed the mathematical model for the generation of the SoA, their model includes some issues such as (i) inconsistency with the comparator model and (ii) blind acceptance of the hypothesis that humans solve inference problems by the Bayes' rule. We addressed these problems by building a mathematical model to provide the experimentally testable hypothesis and by verifying the hypothesis through participant experiments in this study.

In summary, our contribution is divided into two parts: (1) a contribution related to the construction of mathematical models and (2) a contribution related to experimental validation. The contributions related to the construction of mathematical models are (i) pointing out that statistical models and learning algorithms should be considered separately, (ii) the actual construction of a mathematical model using the SGD algorithm as a comparison object for the mathematical model using the Bayesian estimation algorithm, (iii) formulating the likelihood as the SoA, and (iv) the experimental hypothesis that the two proposed mathematical models behave differently in an experimental task where iterative learning is required. Through the participant experiments, we reported supportive evidence that the human subjects updated their prediction models and that the SoA transformed accordingly: the subjects adapted to the visual stimuli representing the result of the action execution even though with temporal biases (Figure 6C). Moreover, our results suggested that the learning algorithm behind the temporal bias adaptation should be described by the Bayes' rule rather than the SGD algorithm (Figure 6D).

We call attention to three limitations of the current study. First, our experimental verification of the theoretical hypothesis was done on only one experimental task (the sense of agency task; Keio method). We need further verification using the variety of the sense of agency tasks to indicate the generalizability of our hypothesis. As our experimental task with beep-referenced temporal biases causes individual differences in reaction time, we needed to take individual differences into account (cf. section 2.5.2). In addition, one could also address that the beep-referenced temporal biases and the button-press temporal biases are qualitatively different experiences for the subjects. Thus, further verification using the other SoA tasks is important. It would also be useful to note that the sense of agency task (Keio method) was originally proposed for evaluating the degree of the schizophrenia patient's SoA (Maeda et al., 2012, 2013). If we could overcome the shortcomings related to the complicated procedures as noted above, we expect our results to be helpful for future schizophrenia research.

Second, we analyzed the experimental data under the assumption that the statistical model for the temporal bias is the Gaussian distribution. While the assumption seems reasonable, note that this is an assumption that simplifies the analysis. We do not exclude all other probability distribution possibilities. In particular, in order to apply our theory to the other tasks (e.g., higher-dimensional prediction problems), the other probability distribution would be suitable. We need to test our theory with other tasks to show that the theory is not limited to the specific probability distribution.

Third, we previously mentioned the limitation associated with this research based on its use of a mathematical model. As Box (1976) has pointed out, we should remember that "all models are wrong" because a model is just an approximation of reality. A traditional guideline for designing a useful model is to illustrate reality as a simple but evocative representation (Box, 1976). Although we followed this guideline to propose the Gaussian model in this paper, a drawback of our approach is that the model was not selected in any quantitative way. Specifically, we did not quantitatively assess the adequacy of the Gaussian assumption and several options for posterior inference, i.e., Markov chain Monte Carlo methods and variational inference methods (Bishop, 2006). Future planned work in this area includes an assessment of our model in comparison to other models using different options according to model selection methods such as information criteria, Bayesian model selection, cross-validation, and norm regularization. (See Ding et al., 2018 for an extensive overview of the selection methods.)

It is useful to discuss the relationship between our model and the model proposed by Legaspi and Toyoizumi (2019) who proposed the Bayesian cue integration model and defined the SoA on the basis of their model. Their definition of the SoA is the maximum value of the posterior distribution. There are at least two important differences between our model and their model. First, our definition of the SoA is the likelihood value that reflects the discrepancies between the prediction and the actual outcome. Second, our model is not intended for the cue integration problem. With respect to the first argument, namely the definition of SoA, it is not that either of the definitions are wrong, but both of them could be right. As Synofzik et al. (2008) pointed out, the SoA is not a single sense but a hierarchical and multilayered sense. Indeed, if we were to adopt a Bayesian model, their definition, i.e., the maximum value of the posterior distribution, could also be defined in our mathematical model. With respect to the second argument, our mathematical framework does not exclude the model for the cue integration problem. Our framework is easy to extend for modeling the cue integration problem if we replace the statistical model appropriately. We hope that the theoretical foundation will continue to be built in the future.

The way in which the probabilistic nature of the SoA can play a key role in the learning process deserves discussion. In its simplest form, the sense of agency originates from the time domain (Gallagher, 2000). In other words, the perception of the onset of the intentional action and the external event gives rise to the perception of the time interval and the SoA is attributed to the perceived time intervals. However, the perception of the time interval is subject to the inevitable noise of sensing and observation. Thus, observable variables should be treated as stochastic variables rather than as deterministic ones. One of the realistic strategies for predicting an event under the uncertainty of sensory information is to infer its distribution from past experiences and thus make a probabilistic prediction.

Perception of time plays a fundamental role in human perception, cognition and action, which is essential for everyday activities and survival. Rhodes (2018) systematically discussed a variety of mathematical models that can predict that the brain uses temporal expectations to bias perception in a way that stimuli are “regularized,” i.e., stimuli look like what has been seen previously. This indicates that the perception of time is subject to various contextual distortions. For example, when observers are presented with various intervals of different lengths and are subsequently instructed to reproduce each interval, they tend to overestimate the duration of short intervals and underestimate that of long intervals (Jazayeri and Shadlen, 2010; Shi et al., 2013). This was reported as a type of “central-tendency” effect, i.e., human subjects migrate their estimates of duration toward the mean of exposed intervals (Rhodes, 2018).

Time is perceived over multiple scales from millisecond scales to interval scales (seconds to minutes), and circadian scales, and these scales should be simulated by different computational and neural mechanisms (Rhodes, 2018). In contrast to the explicit perception of time intervals at a scale of a few seconds, our finding of Bayesian updates in the learning process focused on time perception at a subconscious level (the millisecond scale), which is directly associated with the generation of the SoA. It would be interesting to study the effects of time perception on the SoA at various time scales.

## Data Availability Statement

The datasets presented in this article are not readily available because we need the approval of our institutional ethical committee for the transfer of the dataset to other institutions. Requests to access the datasets should be directed to the corresponding author (shiroyano@ieee.org).

## Ethics Statement

Studies involving human participants were reviewed and approved by Keio University, Tokyo University of Agriculture and Technology. The patients/participants provided their written informed consent to participate in this study.

## Author Contributions

SY mainly designed the theoretical framework of this study. YH and TK supported the work. TM and HI designed the experimental setup. SY and YM performed the analysis of the experimental data. All authors contributed to writing the paper.

## Conflict of Interest

The authors declare that the research was conducted in the absence of any commercial or financial relationships that could be construed as a potential conflict of interest.

## Footnotes

^1^Our Python code for this analysis is available to all at https://github.com/yshlo/gf_curvefit.
